# Enhanced Tunability Achieving at Low Permittivity and Electric Field in (Ba_0.91_Ca_0.09_)(Sn_x_Zr_0.2__−__x_Ti_0.8_)O_3_-2 mol% CuO-1 mol% Li_2_CO_3_ Ceramics

**DOI:** 10.3390/ma16155226

**Published:** 2023-07-25

**Authors:** Bo Wang, Le Zhao, Xiuhuai Jia, Pan Yang, Shihui Yu

**Affiliations:** 1 Department of Electrical Engineering and Automation, Luoyang Institute of Science and Technology, Luoyang 471023, China; 2 School of Microelectronics, Tianjin University, Tianjin 300072, China

**Keywords:** ferroelectric varactors, permittivity, impedance matching, oxygen octahedron

## Abstract

Ferroelectric varactors should have high tunability at low permittivity and a working electric field to obtain better impedance matching and stable tunability. In this work, (Ba_0.91_Ca_0.09_)(Sn_x_Zr_0.2−x_Ti_0.8_)O_3_-2 mol% CuO-1 mol% Li_2_CO_3_ (abbreviated as BCSZT100x, x = 0.05, 0.10, 0.15 and 0.20, respectively) are prepared to achieve high tunability at low permittivity and a working electric field. The tunable mechanisms are investigated based on crystal structure, micro-morphology and the permittivity-temperature spectrum. The results show that the shrink of oxygen octahedron and weaker interaction force between Sn^4+^ and O^2−^ make BCSZT5 ceramic have a higher tunability value of 26.55% at low permittivity (1913) and a working electric field (7.3 kV/cm). The tunability value of BCSZT5 ceramic increases by 58%, while its permittivity decreases by 25%, compared with x = 0. Those advantages make BCSZT5 ceramic have substantial application prospects in varactors.

## 1. Introduction

With the rapid development of The Internet of Things, 5G networks and autonomous traffic, the frequency spectrum has been scarce; it requires the frequency, bandwidth and phase of front-end devices (such as antennas, filters and phase shifters) to be regulated [1,2,3,4]. It is a good strategy to fabricate front-end devices using varactors whose capacitor can be expediently controlled by external magnetic field, electric field or mechanical force [5,6,7,8]. The utilization of varactors not only reduces the volume and manufacturing cost of communication equipment, but also makes communication equipment have the ability of multi-band scanning and transmitting signals. Taking mobile phones as an example, it is usually required to work in multiple communication modes synchronously, such as the global positioning system, Bluetooth and wireless network [9]. It requires to integrate different antennas for corresponding application, and there is no signal interference between those antennas. The requirements increase the volume and manufacturing cost circuits. If varactors are used, the number of discrete transceivers can be significantly reduced, and multi-functions can be achieved at a lower cost.

According to the used technologies and materials, varactors can be divided into micro-electromechanical systems (MEMS) varactors, semiconductor varactors, ferrite varactors and ferroelectric varactors [10,11,12,13]. Among those varactors, ferroelectric varactors have attracted tremendous attention due to advantages of large power handling, high-tuning speed, long switching lifetime and so on [14]. The large nonlinear dielectric responses and lower dielectric loss of ferroelectric materials supports the stronger signal control capability. Usually, the nonlinear dielectric responses can be evaluated by the parameter tunability *K*, which reflects the relative variation of permittivity (ε(E)) in the electric field (K=(1−ε(E)/ε(0))×100%, ε(0): permittivity at zero electric field). It can be seen that large ε(0) is helpful to achieve high tunability. However, varactors should have low permittivity in order to achieve a better impedance matching in circuit, which goes against the high tunability [15,16]. To achieve high tunability at low permittivity, many works improve the working electric field. Zhai et al. reported a higher tunability value of 32% at low permittivity (875) in Ba_0.4_Sr_0.6_TiO_3_-ZnAl_2_O_4_ composite ceramics when electric field strength was 30 kV/cm [17]. Zhang et al. achieved a tunability value of 17.3% and low permittivity of 830 at 30 kV/cm in 30 wt% BaCuSi_2_O_6_-70 wt% Ba_0.55_Sr_0.45_TiO_3_ composite ceramics [18]. Though many works have achieved high tunability at low permittivity, the working electric field is usually more than 15 kV/cm [19,20]. The high-working electric field goes against the stability of tunability due to the influence of polar nano-regions (PNRs) at a repeating electric filed. Yang et al. reported that the growth of PNRs was related to the electric field [21]. However, the contribution of polar nano-regions (PNRs) plays an important role on tunability. When a higher repeating electric field is applied, the stability of tunability is brittle. Xu et al. reported that the tunability of BZT ceramics deteriorates ~3% percent at 20 kV/cm, only after two cycle numbers [22]. With increasing cycle numbers, the degradation rate of tunability increases. The similar tendency also has been found in (Ba_0.6_Sr_0.4_)TiO_3_ ceramics [23]. Thus, achieving high tunability at low permittivity and a working electric field is crucial for the application of ferroelectric varactors in antennas, filters and phase shifters. However, some attention is necessary to investigate reducing the working electric field.

In our previous work, (Ba_0.91_Ca_0.09_)(Zr_0.2_Ti_0.8_)O_3_-2 mol% CuO-1 mol% Li_2_CO_3_ ceramic (abbreviated as BCZT-CL) can achieve a tunability value of 16.76% at a low-working electric field (7 kV/cm) [24]. But the higher permittivity (2634) is adverse to impedance matching and impedes its applications. Tao et al. investigated the dielectric properties of Ba(Ti_1−x_Sn_x_)O_3_ [25]. Sn doping can make off-centre ion displacements unstable. The unstable off-centre ion displacements are expected to enhance the tunability. Thus, (Ba_0.91_Ca_0.09_)(Sn_x_Zr_0.2−x_Ti_0.8_)O_3_-2 mol% CuO-1 mol% Li_2_CO_3_ (abbreviated as BCSZT100x, x = 0.05, 0.10, 0.15 and 0.20, respectively) are prepared to achieve high tunability at low permittivity and a working electric field. When the electric field strength is 7.3 kV/cm, BCSZT5 ceramics have a higher tunability value of 26.55% and lower permittivity value of 1913. The tunability value of BCSZT5 ceramics increases by 58%, while its permittivity decreases by 27%, compared with BCZT-CL ceramics. Those advantages make BCSZT5 ceramics have significant application prospects in varactors. Rigaku Corporation, Japan

## 2. Experimental Procedures

BCSZT100x ceramics were obtained via solid-state sintering using the preparation process reported in our previous work [26]. All mixed raw materials (TiO_2_, ZrO_2_, BaCO_3_, CaCO_3_ and SnO_2_) were calcined at 900 °C for 3 h to obtain pre-synthesis BCSZT powders. Then, 2 mol% CuO and 1 mol% Li_2_CO_3_ were added into pre-synthesis BCSZT powders. BCSZT100x ceramic greens were sintered at 1100 °C for 3 h. Rigaku D/MAX-2500 X-ray diffractometer (XRD, Rigaku Corporation, Tokyo, Japan) and Helios NanoLab 460HP scanning electron microscopy (SEM, Thermo Fisher Scientific, Waltham, MA, USA) were used to investigate the crystal structure and micro-morphology of BCSZT100x ceramics, respectively. Permittivity (*ε*), dielectric loss (tan δ) and tunability (*K*) was measured at 1 kHz using a HP 4278A capacitance meter equipped with a HM27004H C-T-V conversion device.

## 3. Results and Discussion

**Figure 1:**
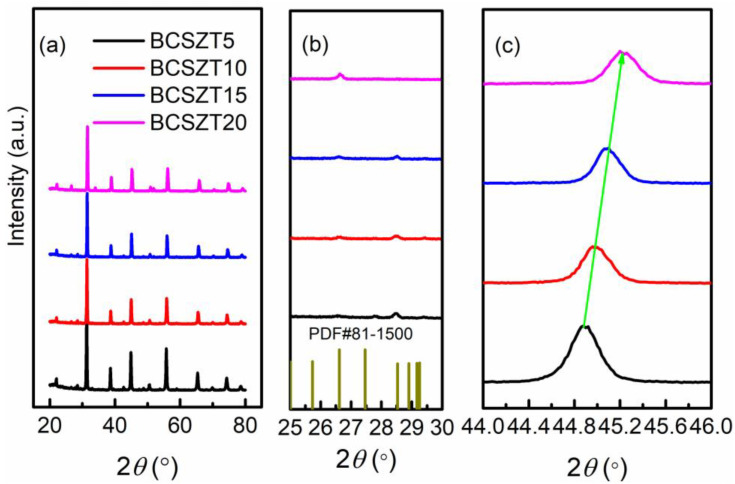
Powder XRD pattern of BCSZT100x ceramics at (**a**) 20~80°, (**b**) 25~30° and (**c**) 44~46°. The dark yellow lines shown in (**b**) are the diffraction peaks of CaZrTi_2_O_7_ (PDF#81-1500) and green lines shown in (**c**) is the moving direction of diffraction peaks.

Figure 1 shows the powder XRD pattern of BCSZT100x ceramics. According to the measured results shown in Figure 1a and the powder diffraction file of JCPDS No. 75-0213, BCSZT100x ceramics sintered at 1100 °C are cubic phase perovskite structures, and the heterodox peaks appearing at the 2θ diffraction angle, ranging from 25° to 30° (shown in Figure 1b), may be the diffraction peak of CaZrTi_2_O_7_ (PDF#81-1500). Figure 1c shows the diffraction pattern of the 2θ diffraction angle between 44° and 46°. Obviously, with the increase of Sn^4+^ content, the diffraction peaks of BCSZT100x ceramics move to a higher angle, indicating the reduction in cell volume of BCSZT100x ceramics. The ionic radius is responsible for the phenomenon. When Sn^4+^ replaces Zr^4+^ to occupy B-site, the smaller ionic radius of Sn^4+^ (0.69 Å), compared with that of Zr^4+^ (0.72 Å), shrinks the lattice, resulting in the shift of diffraction peak to higher angle [27,28].

**Figure 2:**
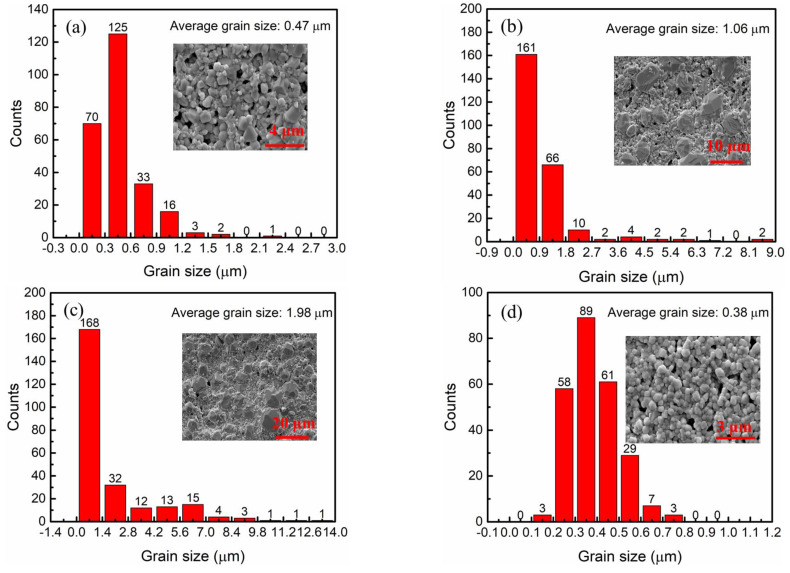
The grain size distribution of BCSZT100x ceramics: (**a**) BCSZT5, (**b**) BCSZT10, (**c**) BCSZT15 and (**d**) BCSZT20.

Figure 2 shows the grain size distribution of BCSZT100x ceramics, and the corresponding SEM results are illustrated. According to Figure 2, it can be seen that the grain size of BCSZT100x ceramics can be effectively regulated by controlling the content of Sn^4+^. As shown in Figure 2a, the grain sizes of BCSZT5 ceramics ranges from 0.15 µm to 1.65 µm, and there are many pores. An average grain size value of 0.47 µm was obtained. When x increases to 0.10, the average grain size of ceramics increases to 1.06 µm, but the grain size distribution range is significantly larger than that shown in Figure 2a. When x further increases to 0.15, the grain size of ceramics ranges from 0.7 µm to 13.3 µm, the average grain size increases to 1.98 µm, and a small number of grain sizes exceed 10 µm. When x is 0.20, the grain size of ceramics ranges from 0.15 µm to 0.75 µm, and the internal grain size is uniformly distributed with an average grain size of 0.38 µm. Based on the above results, the following conclusions can be drawn: the average grain size of BCSZT100x ceramics can be effectively improved by doping with Sn^4+^ properly, but the grain size distribution will be uneven.

**Figure 3:**
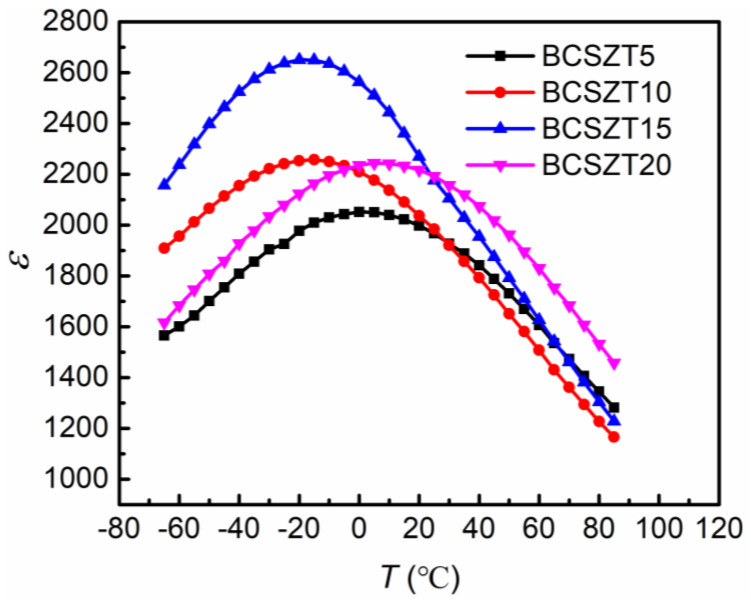
The temperature-dependent permittivity of BCSZT100x ceramics.

Figure 3 shows the variation of the temperature-dependent permittivity of BCSZT100x ceramics. As x gradually increases from 0.05 to 0.15, the Curie temperature gradually moves towards low temperature, and the maximum dielectric constant gradually increases. However, when x increases to 0.20, the Curie temperature of BCSZT20 ceramics increases and the maximum dielectric constant decreases. The above phenomenon is consistent with the conclusion obtained by Wu et al. in (Ba_0.98_Ca_0.02_)(Ti_0.94_Sn_0.06−x_Zr_x_)O_3_ ceramics [29]. In addition, it can be seen from Figure 3 that the permittivity of BCSZT100x ceramics can be significantly improved by doping an appropriate amount of Sn^4+^. The same phenomenon has also been found in (Ba_0.8_Sr_0.2_)(Ti_1−x_Sn_x_)O_3_ and (Ba_0.95_Ca_0.05_)(Ti_0.92_Sn_0.08−x_Zr_x_)O_3_ ceramics [30,31]. The change of permittivity may be mainly affected by grain size. A large number of grain boundaries induced by smaller grain sizes in ceramics can impede the movement of domain, leading to the deterioration of permittivity [32,33]. According to the SEM results, the largest average grain sizes of BCSZT15 ceramics support the highest permittivity. Moreover, the depressed and dilated permittivity of BCSZT100x ceramics suggest the existence of diffused phase transition (DPT) in ceramics. For BaTiO_3_-based ceramics, the DPT depends on the distribution of both A site and B site ions [34,35,36]. The random occupancy of A site or B site ions is in favour of the formation of nonuniform Coulomb field, which facilitates the generation of polar nano-regions (PNRs). However, different PNRs possess diverse local dielectric properties, thus a compressed and dilated permittivity peak can be obtained readily. When A or B sites are occupied by two different ion types, the difference in electronegativity and ionic radii between two different ion types accelerates the formation of PNRs, resulting in the appearance of a more compressed permittivity peak. According to results reported in BaTiO_3_ and (Ba_0.85_Ca_0.15_)(Zr_0.10_Ti_0.90_)O_3_ ceramics, smaller grain sizes also enhanced the diffused phase transition due to the grain size effect [37,38].

**Figure 4:**
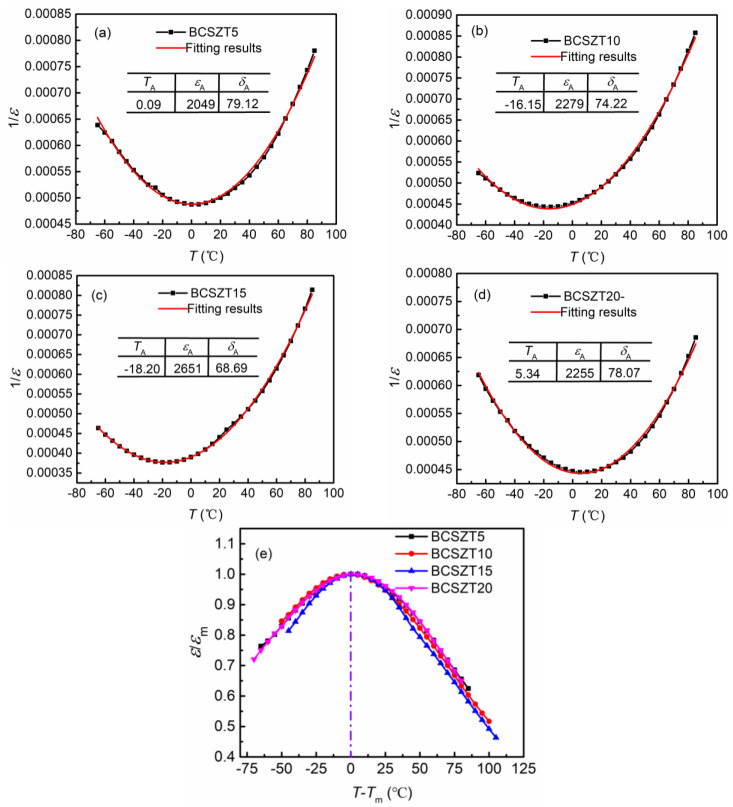
(**a**–**d**) The fitting results using Equation (1) and (**e**) the normalized of permittivity near Cuire temperature.

The DPT of ceramics can be discussed using empirical Lorenz formula [39]:

(1) $$\frac{\varepsilon_{A}}{\varepsilon }=1+\frac{{\left(T-T_{A}\right)}^{2}}{2\delta_{A}{}^{2}}$$

where diffusion coefficient $\delta_{A}$ shown in Equation (1) represents the degree of DPT and $\varepsilon_{A}$ represent the permittivity at temperature $T_{A}$. The fitting results about the temperature-dependent permittivity are shown in Figure 4a–d) and corresponding parameters are listed. Obviously, the diffusion coefficient $\delta_{A}$ of BCSZT100x ceramics has little difference, indicating that there is no significant difference in the degree of DPT. In order to more intuitively compare the degree of DPT of different ceramics, the measured results in Figure 3 are normalized and shown in Figure 4e. The coincident normalized curves indicate that doping Sn^4+^ in BCZT ceramics cannot affect the DPT in our work, which is consistent with the result obtained by Equation (1). This phenomenon contradicts the result reported in Ba(Zr_0.2_Ti_0.8_)_1−x_Sn_x_O_3_ ceramics. Fu et al. found that the degree of DPT in Ba(Zr_0.2_Ti_0.8_)_1−x_Sn_x_O_3_ ceramics became stronger with increasing Sn^4+^ content [40]. In our work, BCSZT100x ceramics are sintered at a low temperature (1100 °C), resulting in the insufficient grain growth, uneven grain size distribution and the smaller average grain size. Thus, the grain size effect makes the DPT of BCSZT100x ceramic cannot been observed significantly.

**Figure 5:**
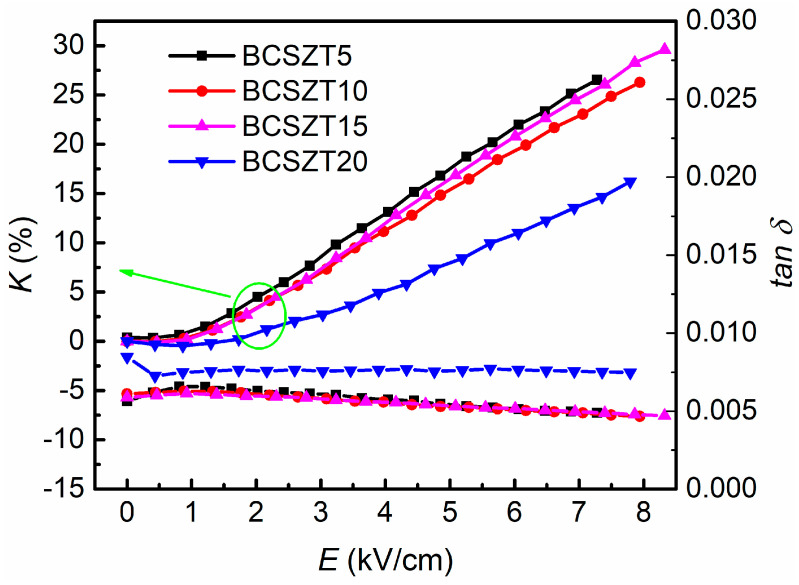
The tunability and dielectric loss of BCSZT100x ceramics.

Figure 5 shows the tunability and dielectric loss of BCSZT100x ceramics under different electric field strengths measured at room temperature. It can be seen that BCSZT5 ceramics have the largest tunability but the smallest for BCSZ20 ceramic under the same electric field intensity. According to Figure 3, it can be seen that the permittivity of BCSZT5 ceramics at room temperature (1913) is smaller than that of BCSZT20 ceramics (2192). Therefore, the tunability of BCSZT20 ceramics should be greater than that of BCSZT5 ceramics according to the previous study. It is obviously inconsistent with the measured results. For BaTiO_3_ based ceramics, tunability are affected not only by grain size, but also by PNRs and oxygen octahedron. Ren et al. studied the tunability of BaTi_0.85_Sn_0.15_O_3_/MgO ceramics [41]. The contribution of polar microregion to tunability is dominant at a lower electric field intensity, while the contribution of B-site ion polarization to tunability is dominant at a higher electric field intensity. According to the results shown in Figure 4, there is no significant difference in the degree of DPT among BCSZT100x ceramics. Therefore, it is inferred that the effect of PNRs on the tunability of BCSZT100x ceramics is basically the same. Meanwhile, Mahmoud et al. studied the tunable properties of (0.95 − x)(Bi_0.5_Na_0.3_K_0.2_)TiO_3_-xSrTiO_3_-0.05 (Ba_0.8_Ca_0.2_)TiO_3_ ceramics [42]. When the temperature was higher than Curie temperature, the permittivity was mainly contributed by the crystal structure. According to XRD results shown in Figure 1, the cell volume of BCSZT100x ceramics decreases and the oxygen octahedron shrinks when increasing the Sn^4+^ content, resulting in the limited movement of B-site ions. It suggests that B-site ions require more energy to generate greater displacement to achieve higher tunability. Therefore, the tunability of BCSZT100x ceramics under the same electric field intensity decreases with increasing Sn^4+^ content. Moreover, the interaction force of B-site ions also plays a key role on the tunability. It can be speculated that the interaction force between Sn^4+^ and O^2−^ is weaker than that between Zr^4+^ and O^2−^ since the melting point of SnO_2_ (1630 °C) is smaller than that of ZrO_2_ (2700 °C) [43,44]. The weaker interaction force between Sn^4+^ and O^2−^ indicates that the movement of B-site ions in the oxygen octahedron will be more sensitive to the electric field after substituting Zr^4+^ with Sn^4+^. Therefore, the tunability of BCSZT5 ceramics (26.55%) is greater than that of BCZT-CL ceramics (16.76%), as reported in our previous work [24].

It can be observed from Figure 5 that BCSZT5 ceramics possess a higher tunability and lower tan δ simultaneously. In order to compare the tunable performance among those ceramics quantificationally, the figure of merit (*FOM*), which takes into consideration *K* and tan *δ* together, is used.

(2) $$FOM=\frac{K}{\tan\delta }$$

**Figure 6:**
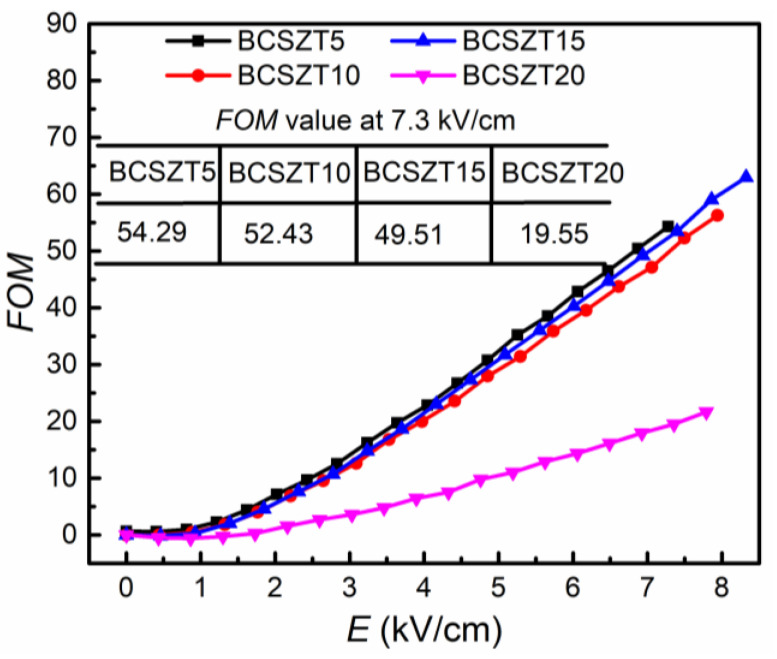
The relationship between *FOM* and electric field strength in BCSZT100x ceramics.

The relationship between the *FOM* value and electric field strength is shown in Figure 6. With increasing Sn^4+^ content, the *FOM* value of BCSZT100x ceramics gradually decreases at the same electric field intensity. When the electric field strength is 7.3 kV/cm, the BCSZT5 ceramics have the highest *FOM* value (54.29) due to their higher tunability (26.55%) and lower tan δ (0.00489), which is higher than that of BCZT-CL ceramics (34.98) [24]. The *FOM* value of BCSZT5 ceramics increases by 58%, while its permittivity decreases by 25%, compared with BCZT-CL ceramics at 7.3 kV/cm. The results show that substituting Zr^4+^ with Sn^4+^ is beneficial to improve the tunable performance. Table 1 shows the tunable performance of other ceramics [45,46,47,48,49,50,51,52]. Obviously, BCSZT5 ceramics not only have a low sintering temperature (1100 °C) and permittivity (1913), but also can achieve high tunability under a lower electric field strength. Those advantages make BCSZT5 ceramics have greater application prospects in varactors.

## 4. Conclusions

BCSZT100x ceramics are prepared to achieve high tunability at low permittivity and a working electric field. The crystal structure, micro-morphology and permittivity-temperature spectrum are used to study the diffused phase transition and tunable mechanisms. XRD results show that the cell of BCSZT100x ceramics shrinks with the increase of Sn^4+^ content because the ionic radius of Sn^4+^ (0.69 Å) is smaller than that of Zr^4+^ (0.72 Å). The decrease of cell supports to the shrink of oxygen octahedron. Meanwhile, SEM results indicate that the average grain size of BCSZT100x ceramics can be effectively improved by doping Sn^4+^ properly, but the grain size is still less than 2 µm. The smaller grain size obtained in BCSZT100x ceramics means that the DPT of BCSZT100x ceramics cannot be observed significantly. It indicates that the tunability of BCSZT100x ceramics are primarily affected by oxygen octahedron. The shrink of oxygen octahedron with increasing Sn^4+^ content suggests that the displacement of B-site ions is suppressed, resulting in the reduction in tunability with increasing Sn^4+^ content. However, the weaker interaction force between Sn^4+^ and O^2−^, which is speculated based on the melting point of SnO_2_ and ZrO_2_, makes the movement of B-site ions in the oxygen octahedron be more sensitive to the electric field. The influence, by shrinking the oxygen octahedron and weaker interaction force between Sn^4+^ and O^2−^, means that BCSZT5 ceramics have a higher tunability value of 26.55% at low permittivity (1913) and a working electric field (7.3 kV/cm). Those advantages make BCSZT5 ceramics have greater application prospects in varactors.

## Figures and Tables

**Table 1:** The tunable performance of other ceramics reported in references.

Samples	Sintering Temperature (°C)	*ε*(0)	Tunability@Working Electric Field	Ref.
Ba_0.5_Sr_0.5_TiO_3_	1400	2500	22.7% (30 kV/cm)	[45]
Ba_0.5_Sr_0.5_TiO_3_	1230	~1746	47% (100 kV/cm)	[46]
Ba_0.55_Sr_0.45_TiO_3_-20 wt% ZnAl_2_O_4_	1400	2362	46.4% (20 kV/cm)	[47]
BaZr_0.25_Ti_0.75_O_3_-10 wt%MgO	1350	1821	38.2% (10 kV/cm)	[48]
Ba_0.6_Sr_0.4_TiO_3_+ 0.8 wt% Li_2_O	900	1900	16.4% (30 kV/cm)	[49]
0.95BaTiO_3_-0.05 CaSnO_3_	1400	~2100	21.15% (30 kV/cm)	[50]
K_0.5_Na_0.5_NbO_3_-0.2SrTiO_3_	1250	1126	17.4% (50 kV/cm)	[51]
0.875BaTiO_3_-0.125Bi(Mg_2/3_Nb_1/3_)O_3_	1250	~1050	<3% (40 kV/cm)	[52]
BCSZT5	1100	1913	26.55% (7.3 kV/cm)	This work

## Data Availability

The data that support the findings of this research are available from the corresponding authors on reasonable request.
